# Multiple Mini Journal Clubs to Improve Malaysian Trainee Psychiatrists' Critical Appraisal Skills

**DOI:** 10.1192/bjo.2024.306

**Published:** 2024-08-01

**Authors:** Jiann Lin Loo, Muhammad Arif Muhamad Rasat, Noor Melissa Nor Hadi, Nicholas Tze Ping Pang

**Affiliations:** 1 Betsi Cadwaladr University Health Board, Wrexham, United Kingdom; 2 Royal Prince Alfred Hospital, Sydney, Australia; 3 Universiti Putra Malaysia, Serdang, Malaysia; 4 Universiti Malaysia Sabah, Kota Kinabalu, Malaysia

## Abstract

**Aims:**

The skill of critical appraisal is mandatory for evidence-based psychiatric practice although the process of learning can be tough for busy psychiatrist trainees. Ironically, reading alone does not translate into skill acquisition. The accessibility to conventional journal clubs may also be limited for doctors working in busy non-academic training centres. Therefore, attending an intensive workshop on critical appraisal skills can be a viable solution. This study elucidated the experience of using an innovative approach, i.e. Multiple Mini Journal Clubs (MMJC), to improve Malaysian trainee psychiatrists’ critical appraisal skills.

**Methods:**

A one-day workshop was conducted for 19 participants who were preparing for MRCPsych Paper B, using the combination of 1) a pre-recorded video lecture with a two-hour question and answer session; 2) three 45-minute stations in a group of three persons to practice critical appraisal of a cross-sectional, a validation, and a randomised controlled study. A standardised approach, i.e. Critical Appraisal in Five Expressed Steps (CAFES), was used by facilitators. CAFES involved asking and answering the following big heading questions while incorporating other standard critical appraisal techniques under each of the headings: 1) What is the research question; 2) Can the research methodology answer the question; 3) Does the result make sense; 4) Are the findings translatable to my setting; 5) How to improve the study if I were to conduct a similar study. Three formative assessments were carried out using Single Best Answer and Extended Matching Items. Qualitative feedback and informed consent were collected.

**Results:**

Hundred per cent of participants agreed that their objective of attending the workshop had been achieved through the MMJC, i.e. learned both the theory and skill of critical appraisal which allowed immediate translation into practice during the MMJC. Nevertheless, there was no statistical difference in participants’ achievement for pre-, mid- and post-workshop formative assessments, i.e. median of 7/25, 7/28, and 8/27 respectively. Positive responses toward MMJC included less performance anxiety in a small group, active interaction, individualised feedback, and fun. The challenges faced included the need for strict time management and a big group of facilitators. Suggestions for improvement included the extension of the workshop duration and breaking up the lecture into several sessions.

**Conclusion:**

Further improvement and re-evaluation of the effectiveness of MMJC is required to optimise learning outcomes.

